# Factors contributing to the recruitment and retention of rural pharmacist workforce: a systematic review

**DOI:** 10.1186/s12913-021-07072-1

**Published:** 2021-10-05

**Authors:** Daniel Terry, Hoang Phan, Blake Peck, Danny Hills, Mark Kirschbaum, Jaclyn Bishop, Kehinde Obamiro, Ha Hoang, Hoang Nguyen, Ed Baker, David Schmitz

**Affiliations:** 1 School Health, Federation University, PO Box 663, Mt Helen, Victoria Australia; 2 Menzies Institute for Medical Research, University of Tasmania, Tasmania, Australia; 3 School of Health, Federation University Australia, Victoria Ballarat, Australia; 4 School of Health, Federation University, Victoria Ballarat, Australia; 5 School of Social Sciences, University of Tasmania, Tasmania, Australia; 6 Pharmacy Board of Australia, Perth, Australia; 7 Western Alliance, Warrnambool, Victoria Australia; 8 Centre for Rural Health, University of Tasmania, Tasmania, Australia; 9 Wicking Dementia Research and Education Centre, University of Tasmania, Tasmania, Australia; 10 Center for Health Policy, Boise State University, Boise, Idaho USA; 11 Department of Family and Community Medicine, University of North Dakota, Grand Forks, USA

**Keywords:** pharmacist, recruitment, retention, systematic review

## Abstract

**Background:**

Recruiting and retaining medical, nursing, and allied health professionals in rural and remote areas is a worldwide challenge, compromising continuity of care and population health outcomes in these locations. Specifically, pharmacists play an essential and accessible frontline healthcare role, and are often the first point of contact for health concerns. Despite several incentives, there remains a maldistribution and undersupply of pharmacists in rural and remote areas across many parts of the world. Although current systematic reviews have focussed on factors affecting pharmacists’ retention generally, literature specifically focused on rural pharmacist workforce in a global context remains limited. The aim of this systematic review is to identify factors associated with recruitment and retention of the pharmacist workforce in rural and remote settings. Better understanding of these contributors will inform more effective interventional strategies to resolve pharmacist workforce shortages.

**Methods:**

A systematic search of primary studies was conducted in online databases, including Medline, Embase, CINAHL, Scopus, Web of Science and PsycINFO, and by hand-searching of reference lists. Eligible studies were identified based on predefined inclusion/exclusion criteria and methodological quality criteria, utilising the Critical Appraisal Skills Programme (CASP) and Good Reporting of A Mixed Methods Study (GRAMMS) checklists.

**Results:**

The final review included 13 studies, with quantitative, qualitative, or mixed methods research design. Study-specific factors associated with recruitment and retention of pharmacists in rural practice were identified and grouped into five main themes: geographic and family-related, economic and resources, scope of practice or skills development, the practice environment, and community and practice support factors.

**Conclusions:**

The results provide critical insights into the complexities of rural recruitment and retention of pharmacists and confirms the need for flexible yet multifaceted responses to overcoming rural pharmacist workforce challenges. Overall, the results provide an opportunity for rural communities and health services to better identify key strengths and challenges unique to the rural and remote pharmacist workforce that may be augmented to guide more focussed recruitment and retention endeavours.

**Supplementary Information:**

The online version contains supplementary material available at 10.1186/s12913-021-07072-1.

## Background

The recruitment and retention of medical, nursing, and allied health professionals in rural and remote areas remains a worldwide challenge, compromising continuity of care and population health outcomes [1, 2]. This is despite increases in training, funding and targeted programs aiming to attract and retain pharmacists [2]. A number of policy responses have led to some gains, such as communities ‘growing their own’ training opportunities that provide rural clinical experiences and rural exposure [3–7]. However, inadequate recruitment and retention of health professionals continues to be problematic, which further impacts the health and wellbeing of rural and remote populations [3, 8–10].

Although current health workforce research or efforts in improving health outcomes often centre on medical practitioners and nurses, pharmacists also play a vital role in the delivery of health care in both primary and secondary settings. Pharmacists in the community critically support equity of access to health services, particularly for those living in rural and remote areas. Beyond medication dispensing, stewardship and safety, pharmacists, community pharmacists, provide direct, accessible, and frontline healthcare for their communities. They are often the first point of contact in rural communities, playing a critical role in the triage of care and referrals of community members to other health professionals [11–13]. In many instances, the pharmacist is the only health professional in a rural or remote community, and pharmacies often serve as the local hub for community healthcare services, particularly for older people and those who are acutely unwell [2, 14, 15].

Despite geographic, financial, and cultural diversities between countries, there remains a maldistribution and undersupply of the pharmacist workforce in rural and remote areas in many parts of the world [2, 16]. Due to this maldistribution, government initiatives have been established to encourage recruitment and retention of rural pharmacists. One such initiative is the provision of rural pharmacy training packages for pharmacy students, often as tailored interprofessional placements [5, 17]. Current research and initiatives tend to focus on undergraduate pharmacy students with few studies targeting the post-registration pharmacist workforce. This limits our understanding of whether the exposure to rural practice from initiatives aimed at registered pharmacists leads to long-term impacts on the recruitment and retention of pharmacists in rural settings [2].

Beyond current empirical studies, two literature reviews have been undertaken to understand these challenges and what drives healthcare practitioners, including pharmacists, to consider rural practice and drivers of rural recruitment and retention [2, 16]. First, a systematic review of the global pharmacist workforce was undertaken to identify the main factors affecting pharmacist retention, regardless of the setting. These included job satisfaction, working conditions, role and responsibilities, policies, training and workload [16]. Second, a scoping review conducted by Obamiro and colleagues [2] focussed on identifying defined strategies to increase the rural and remote pharmacist workforce, or factors associated with the retention of pharmacists in rural or remote areas. Although insightful, the evidence was limited to Australian contexts. In addition, the retention factors identified by Obamiro et al. [2] were based on five studies only, and the overall findings highlighted personal, community, and workplace factors were important in impacting recruitment, retention, and the perception of pharmacist in being a good fit within rural contexts.

Further, the work of Carvajal [18] highlights that, globally, the rural and remote pharmacist workforce shares common challenges that are likely interconnected and drive workforce decision-making. However, a comprehensive understanding of the factors that drive the recruitment and retention of pharmacists in rural practice from an international perspective remains lacking. Based on the limited published literature, Carvajal [18] provided a theoretical framework for the interpretation of the challenges identified in studies across the world focused on the pharmacist workforce and comprise such factors as personal characteristics, human capital, job-related preferences, perceptions, and rigidities. A deeper understanding of these factors in a global context requires further investigation to determine the specific barriers and facilitators experienced by pharmacists. This remains essential to identifying, developing, and implementing tailored programs designed to increase the rural and remote pharmacist workforce in meeting the needs of their communities.

Within this context, the aim of this systematic review was to comprehensively identify the factors associated with recruitment and retention of the pharmacist workforce in rural and remote settings. Findings from this review may inform more effective interventional strategies to resolve pharmacist workforce shortfalls.

## Methods

A systematic examination of the primary research literature examining recruitment and retention factors for the pharmacist workforce in rural and remote settings (hereafter termed ‘rural pharmacist workforce recruitment and retention’). The Cochrane handbook for systematic reviews of interventions – 2nd edition [19] was used to guide the identification, extraction and evaluation of data in included studies. The objectives, analysis methods and inclusion/exclusion criteria were developed and documented to ensure accurate and complete reporting of findings, as outlined by the Preferred Reporting Items for Systematic reviews and Meta-Analyses (PRISMA) 2020 statement [20] (Additional file 1).

### Search strategies

The search of the literature was conducted on April 9, 2021 using Medline, Embase, CINAHL, Scopus, Web of Science, and PsycINFO. Databases were initially searched for all potentially relevant studies using the title, abstract and full texts fields. Key terms were adapted to each database’s specific requirements, with a search strings that included: “rural*”, “remote*”, “regional*”, “location”, “recruit*”, “retain*”, “retention*”, “turnover”, “leave”, “remain”, “intend*”, “intention”, “decision*”, “pharmac*”, “health*”, “workforce”, and “profession*”. No limit in date of publication was included when initially searching each database. Hand searching and reviewing of reference lists were also employed to identify additional relevant studies. The review also undertook an initial examination of grey literature, such as government reports, issue papers, policy statements, and Doctor of Philosophy (PhD) theses.

### Inclusion and exclusion criteria

This systematic review included original studies, either quantitative, qualitative, or mixed methods, designed to identify factors associated with rural pharmacist workforce recruitment and retention. Study participants were pharmacists at all stages of careers, including pharmacy graduates/novices, where relevant. Final year undergraduate pharmacy students were considered for inclusion if the focus of the study was on intention to practise rurally. Mixed professions (e.g., Allied health), including pharmacists, were considered for inclusion if the factors associated with rural pharmacist workforce recruitment and retention were specified and clearly demarcated between professions. Both community and hospital settings were included. Once papers were searched, the publication date for articles were limited to 1996 onwards. This coincides with a global shift in health workforce preparation with multi-professional university department of rural health beginning to appear. Their focus being on providing education and training in more rural centres, in order to attract health professionals to practise in rural and remote communities [21, 22].

Articles were limited to peer-reviewed empirical studies and were excluded if they were systematic reviews, discussion papers or protocols of in-progress studies. Studies were excluded if they did not focus on rural pharmacist workforce recruitment and retention, or those that focused purely on pharmacy students who were not in their final year of undergraduate training. It is recognised that locum support remains an essential element to strategies for rural pharmacies. However, studies with a sole focus on the employment of sessional pharmacists were excluded, as the focus of this review is on longer term recruitment and retention strategies. Due to challenges in ensuring translation qualities, only full-text articles in English were included in this review.

### Data screening, selection, and extraction

The studies retrieved from all sources were exported to EndNote (Version X9). Two reviewers (HP and DT) worked together in screening and selecting studies after the removal of duplicates. The reviewers independently undertook a blind screening of the titles and abstracts of all studies to exclude obviously irrelevant studies and identify a list of potentially relevant studies. The same two reviewers then independently undertook a blind assessment of the full texts against the inclusion criteria. Differences or disagreements between the two reviewers were resolved through discussion and consultation with another reviewer (BP) until consensus was reached. One reviewer (HP) also performed a hand search of the reference lists from the included studies to identify other eligible studies, followed by discussion with the reviewer team. At each stage, the reasons for inclusion and exclusion were clearly documented.

Data from the included studies were extracted and documented in a data extraction form. Important extracted factors included research design, participants, time points, outcome measures, and list of contributing factors. Correspondence with the authors of some specific articles were made by one reviewer to request more detailed information about methods and statistical results.

### Methodological quality assessment procedure

Using a criteria checklist aligned with the Cochrane guidelines (Higgins et al., 2019), two reviewers independently assessed the methodological quality of each study. The scoring of publications was conducted using the Critical Appraisal Skills Program (CASP) tool for qualitative and cross-sectional research [23], and Good Reporting of A Mixed Methods Study (GRAMMS) for ‘mixed methods’ research [24].

These quality assessment criteria were related to selection, performance, attrition, detection, and reporting; the five common types of research bias. For each of the methodological quality criteria, the studies were rated as (+) criterion met, (-) criterion not met, (u) unknown if the criterion was met or not, and (n/a) not applicable. The overall scoring of the CASP qualitative and cross-sectional studies were a maximum of 11.0 and 20 respectively, while the scoring of the GRAMMS was a maximum of 6.0 [23, 24]. Two reviewers (HP and DT) discussed the quality assessment results and consulted with a third reviewer (BP) as needed.

### Data analysis

Informed by the approach to qualitative systematic review outlined by Sandelowski et al. [25], the data extraction was undertaken by two reviewer (HP and DT) who extracted all data using Microsoft Word. Following a modified version of the process outlined by Colaizzi [26] each reviewer (HP and DT) independently read and re-read each article identified in order to formulate significant statements, meaning, as well as the interpretation, ideas, accounts and assumptions of what the findings presented by the authors of each identified papers represented. Reviewers then shared and discussed the interpretation of the articles resulting from the independent review. Common or recurring patterns in the significant statements and meanings were aggregated and formulated into thematic representations of study-specific factors.

The quantitative approach for mixed research synthesis was informed by Voils et al. [27] and Crandell et al. [28], however, the vast heterogeneity of research articles, hypotheses, research questions, methodology, outcome measures, and findings of each individual study precluded undertaking in-depth meta-analysis. Due to reporting inconsistencies between studies, sensitivity analyses including sub-group analysis by study design (qualitative, quantitative, and mixed methods) were also not undertaken. Data (continuous, categorical, or qualitative) were therefore synthesised into five themes to undertake data analysis, as guided by the work of Schmitz et al. [29]. Due the diversity and quality of data extracted only descriptive statistics of the data and key findings from each study were analysed. Again, each reviewer (HP and DT) independently examined each article in order to identify significant findings and meaning from the quantitative data, while developing an interpretation of what the collective data were presenting from the identified papers.

Previous research by Cosgrave [30], identified three whole-of-person domains provided insight into understanding and informing the complex interplay between the various personal, social, organisational, and spatial factors that contribute to health professional’s retention. However, within this context, the framework by Cosgrave [30], although informative, at this juncture did not allow a more detailed breakdown or nuanced understanding of the essential elements associated with rural pharmacist workforce recruitment and retention.

## Results

**Fig. 1:**
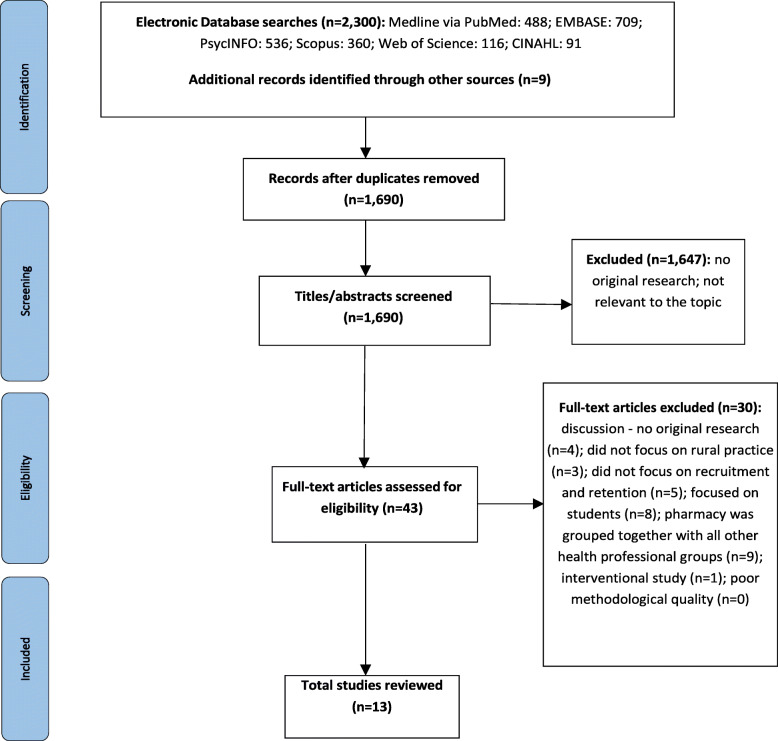
Systematic review flow chart.

After removing duplicates, the systematic search yielded 1,690 potentially relevant publications. After screening out those that did not meet the inclusion criteria, a total of 43 articles were identified and full texts were retrieved. An additional 30 studies were excluded from the review due to not being original research nor focusing rural pharmacist workforce recruitment and retention (Fig. 1).

Overall, 13 studies met the criteria for inclusion and were relevant to the research aim. The studies included six quantitative [6, 7, 31–34], four qualitative [15, 35–37] and three mixed methods studies [38–40]. All 13 included studies were cross-sectional and had at least moderate methodological quality (Tables 1, 2 and 3). The quality score of the quantitative studies ranged from 12 to 20, with almost all (83 %) having high-quality methodology. Similarly, three qualitative publications had high scores (9.0) of methodology quality while the remaining study was of moderate methodology quality (8.0). All three publications using mixed methods had a score of 3.0 or higher, and they were considered to be of moderate methodological quality.

**Table 1:** Methodological quality assessment of quantitative articles using Critical Appraisal Skills Programme (CASP) checklist - cross-sectional studies

Author (Year), Country	A	B	C	D	E	F	G	H	I	J	K	L	M	N	O	P	Q	R	S	T	Total	Quality of research paper
Glasser, 2006; US	+	+	-	+	-	-	+	+	+	+	+	+	-	-	-	+	+	+	-	-	12	Moderate
Fleming and Spark, 2011; Australia	+	+	+	+	+	+	+	+	+	+	+	+	-	+	+	+	+	+	-	+	18	High
Pearson et al., 2010; Canada	+	+	+	+	+	+	+	+	+	+	+	+	+	+	+	+	+	+	+	+	20	High
Woodend et al., 2004; Canada	+	+	-	+	+	+	-	+	+	+	+	+	-	-	+	+	+	+	-	-	14	High
Ling et al., 2018; New Zealand	+	+	+	+	+	+	-	+	u	+	+	+	-	-	+	+	+	+	-	+	15	High
Daniels et al., 2007; the US	+	+	+	+	+	+	-	+	+	+	+	+	-	-	+	+	+	+	-	+	16	High

Quality criteria: A: Were the aims/objectives of the study clear?, B: Was the study design appropriate for the stated aim(s)?, C: Was the sample size justified?, D: Was the target/reference population clearly defined? (Is it clear who the research was about?), E: Was the sample frame taken from an appropriate population base so that it closely represented the target/reference population under investigation?, F: Was the selection process likely to select subjects/participants that were representative of the target/reference population under investigation?, G: Were measures undertaken to address and categorise non-responders?, H: Were the risk factor and outcome variables measured appropriate to the aims of the study?, I: Were the risk factor and outcome variables measured correctly using instruments/measurements that had been trialled, piloted or published previously?, J: Is it clear what was used to determined statistical significance and/or precision estimates? (e.g. p-values, confidence intervals), K: Were the methods (including statistical methods) sufficiently described to enable them to be repeated?, L: Were the basic data adequately described?; M: Does the response rate raise concerns about non-response bias? (negative denotes no any attempt made to quantify the level of non-response by the researchers, and/or the response rate provided is likely to lead to non-response bias), N: If appropriate, was information about non-responders described?; O: Were the results internally consistent?; P: Were the results presented for all the analyses described in the methods?, Q: Were the authors’ discussions and conclusions justified by the results?; R: Were the limitations of the study discussed?, S: Were there any funding sources or conflicts of interest that may affect the authors’ interpretation of the results? (negative denotes no mention of any funding sources or conflicts of interest); T: Was ethical approval or consent of participants attained? (negative denotes no mention of any ethical approval or consent of participants attained); +: yes (criterion is met), −: no (criterion is not met), u: unknown if criterion is met, n/a: not applicable; High-quality paper: Scores ≥ 14, Moderate-quality paper: Scores 8-<14, Low-quality paper: Less than 8.

**Table 2:** Methodological quality assessment of qualitative articles using Critical Appraisal Skills Programme (CASP) checklist

Author (Year), Country	A	B	C	D	E	F	G	H	I	J	Total	Quality of research paper
Allan et al., 2007; Australia	1	1	1	1	1	0	1	1	1	1	9.0	High
Allan et al., 2008; Australia	1	1	1	1	1	0	1	1	1	1	9.0	High
Harding et al., 2006; Australia	1	1	1	1	1	0	1	1	1	0	8.0	Moderate
Hays et al., 2020; Australia	1	1	1	1	1	0	1	1	1	1	9.0	High

Quality criteria: A: Was there a clear statement of the aims of the research?; B: Is a qualitative methodology appropriate?; C: Was the research design appropriate to address the aims of the research?; D: Was the recruitment strategy appropriate to the aims of the research?; E: Was the data collected in a way that addressed the research issue?; F: Has the relationship between researcher and participants been adequately considered?; G: Have ethical issues been taken into consideration?; H: Was the data analysis sufficiently rigorous? I: Is there a clear statement of findings?; J: How valuable is the research?/Recommendations; 1: Yes, 0.5: Unsure, 0: No; High-quality paper: Scores 9.0–10.0, Moderate-quality paper: Scores 7.0-<9.0, Low-quality paper: Less than 7.0.

**Table 3:** Methodological quality assessment of mixed methods articles – Good Reporting of A Mixed Methods Study (GRAMMS)

Author (Year), Country	A	B	C	D	E	F	Total	Quality of research paper
Anzenberger, 2011; Ukraine	0.5	0.5	0.5	0	0.5	1	3.0	Moderate
Smith, 2013; Australia	1	1	1	0	0.5	0.5	4.0	Moderate
Taylor et al., 2019; Australia	0.5	0.5	1	0	0.5	0.5	3.0	Moderate

Quality criteria: A: Describe the justification for using a mixed methods approach to the research question; B: Describe the design in terms of the purpose, priority and sequence of methods; C: Describe each method in terms of sampling, data 13 collection and analysis; D: Describe where integration has occurred, how it has occurred and who has participated in it; E: Describe any limitation of one method associated with the present of the other method; F: Describe any insights gained from mixing or integrating methods; 1: Yes, 0.5: Yes but; 0: No; High-quality paper: Scores 5.0–6.0, Moderate-quality paper: Scores 3.0-<5.0, Low-quality paper: <3.0.

Of the included 13 studies (Table 4), eight (62 %) focused on pharmacists only or reported on recruitment and retention factors associated with practicing rurally among health professionals generally. The latter studies, which provided separate data specifically for pharmacists, included one study from Canada [33] and seven studies from Australia [6, 15, 35–37, 39, 40]. There were two studies reporting factors for final year pharmacy students and recent graduates, with one from New Zealand [34] and the other from Canada [32]. The remaining three studies (n = 2 from US and n = 1 from Ukraine) included pharmacists or final year pharmacy students and/or recent graduates under the umbrella of, and mixed in with, allied health practitioners [7, 31, 38]. It is noteworthy that the research by Glasser and colleagues [31] met the criteria for inclusion, despite study participants being hospital Chief Executive Officers. Although differing slightly, the main aim of the study was the identification of factors associated with rural health workforce recruitment and retention, including pharmacists [31].

**Table 4:** Characteristics of studies included in the review

Author (Year), Country	Design; Time points	Participant group*	Participants	Data collection tools and procedures
Allan et al., 2007; Australia	Qualitative; convenience sampling of pharmacists and social workers; Jul – Aug 2006	1: pharmacists	n = 6 pharmacists and n = 5 social workers (NSW; six rural communities with populations < 5,000)	Initial contact via telephone; information and consent form via mail; Qualitative in-depth semi-structured interview
Allan et al., 2008; Australia	Qualitative; convenience sampling of pharmacists and social workers; Jul – Aug 2006	1: pharmacists	n = 6 pharmacists and n = 5 social workers (NSW; six rural communities with populations < 5,000)	Initial contact via telephone; information and consent form via mail; Qualitative in-depth semi-structured interview
Anzenberger, 2011; Ukraine	Mixed method; quantitative with questionnaires (Jul 2009) and qualitative design; (Sep 2009 – March 2011)	3: mixed	n = 58 pharmacy students in 2 final years in quantitative study; n = 10 volunteer students who were not included in the quantitative investigation and 15 academic and scientific staff in the individual interviews	Participants in the quantitative investigation were recruited randomly from classrooms. They were fully informed about the research and invited to complete a questionnaire. Students for the qualitative investigation were recruited via advertising on the university notice board, and staff-member participants were nominated the second author (a faculty member). The individual interviews were of 60 min duration and conducted in English using a professional translator for the Russian translation
Glasser, 2006; US	Quantitative; time not reported	3: mixed	n = 22 hospital Chief Executive Officers regarding their views on recruitment and retention of rural health workforce, i.e., pharmacists, nurses, physicians, etc.	Mail survey; 2-page questionnaires
Harding et al., 2006; Australia	Qualitative; Jul – Sep 2002	1: pharmacists	n = 12 community pharmacists	Semi-structured in-depth interviews with n = 11 pharmacists and 1 telephone interview. (nationwide)
Hays et al., 2020; Australia	Qualitative; time not reported	1: pharmacists	n = 12 pharmacists (early middle and late career represented)	Semi-structured interviews using purposive non-probability sampling; the interview questions were piloted with two pharmacists and minor changes to language were made
Fleming and Spark, 2011; Australia	Quantitative; Jul – Aug 2009	1: pharmacists	n = 202 early career pharmacists (Victoria) living in Vic 2009; registered with pharmacy Board of Victoria after 1 October 2004; A stratified sample of all 264 rural pharmacists and a random sample of 350 major city pharmacists were taken from the population.	Mail survey with questionnaire; Cognitive interviews, with six pharmacists, were conducted to pre-test the questionnaire
Smith, 2013; Australia	Mixed method; Aug 2009	1: pharmacists	n = 652 pharmacists in quantitative study; n = 143 pharmacists in focus group; n = 83 pharmacists in the semi-structure interview (Victoria)	A qualitative national consultation and a quantitative rural and remote pharmacist workforce survey. Semi-structured interviews (n = 83) and focus groups (n = 15, 143 participants) were conducted with stakeholders with an interest in rural and remote pharmacy, practising rural/remote pharmacists and pharmacy educators, and as well as with peak pharmacy organisations.
Taylor et al., 2019; Australia	Mixed method; time not reported	1: pharmacists	n = 92 pharmacists with 12 survey participants undertaking interviews	A questionnaire and a semi-structured in-depth interviews. The questionnaire was distributed to rural pharmacist networks using a purposive nonprobability sampling method; The invitation to participate was provided via multiple methods including email, newsletter distribution and Facebook posts. Interviews via telephone were conducted with 12 survey participants, who volunteered to contribute
Pearson et al., 2010; Canada	Quantitative; spring 2007	2: pharmacy student/recent graduates	n = 102 graduate pharmacists	Paper-based distribution of questionnaires. The questionnaire was prepared based on findings from the literature and from interviews with graduates from the previous year (n = 12), who also pilot tested a draft questionnaire.
Woodend et al., 2004; Canada	Quantitative; 2001	1: pharmacists	n = 1019 pharmacists	The 8-page survey was mailed to a random sample of 2524 pharmacists living in rural and remote Canadian communities
Ling et al., 2018; New Zealand	Quantitative; time not reported	2: pharmacy student/recent graduates	n = 3121 domestic health professional graduates receiving New Zealand Government Student Loan from 2006–2016: pharmacy (n = 862; 27.6 %)	Exit surveys; online or paper-based format was not specified.
Daniels et al., 2007; US	Quantitative design with questionnaire; time not reported	3: mixed	n = 765 graduates from 12 health professional programs in New Mexico graduated between 1991 and 2002: pharmacy (n = 178; 23.3 %)	Mail survey (7-page survey); The survey was piloted among graduates who were in practice and revised following the pilot and ensuing focus group.
*Participant group: 1: pharmacists (studies reporting factors for pharmacy separately with other health professionals are eligible); 2: pharmacy student/recent graduates, and 3: pharmacists or pharmacy student/recent graduates under the umbrella of and mixed in with Allied health practitioners				

Of the 13 studies, five reported barriers or enablers contributing to pharmacists’ employment or intention to practice in rural settings, or job location decision, which were likely considered as factors associated with recruitment [6, 32, 34, 38, 40]. One study focused on identification of retention factors only [33]. Factors affecting rural pharmacist workforce recruitment and retention were clearly differentiated in two publications [7, 37]. Conversely, this was not the case for the remaining studies, where the factors were defined as enablers or barriers to rural pharmacist practice [15, 31, 35, 36, 39]. A list of study-specific factors is provided elsewhere (Additional file 2 and 3).

Based on the key statements and their meaning from each of the identified studies, the five main recruitment and retention themes or groups of factors, included geographic and family-related factors, economic and resource factors, scope of practice or skills development factors, practice environment factors, and community and practice support factors. Each theme and their respective factors were grouped according to each study (Additional file 4), are summarised in Table 5, and discussed in detail below.

**Table.5:** Lists of factors based on five major themes

Geographic (and family-related) factors	Economic/resources	Scope of Practice/Skills Development	Practice Environment	Community/practice support
1. Family-friendly environment 2. Married with children living at home 3. Rural lifestyle 4. Lived in rural areas/Rural background 5. Spouse or partner with a rural background 6. Spousal/partner satisfaction e.g. education, work, general 7. Desire to return to hometown 8. Familiarity of rural and remote location 9. Family in a rural area 10. Being independent from extended family 11. Size of community 12. Owning a pharmacy in rural area (as a geographic aspect) 13. Good public school system 14. Recreational opportunities/adventure experiences 15. Social and/or cultural activities 16. Opportunities for family members 17. Having family accompany 18. Personal and Social relationships 19. Social and cultural facilities 20. Multiculturalism/Many cultures existing in a community 21. Connection with community members of the same age 22. Availability of public media, communication, and internet 23. Availability of public transport 24. Good transport connections – road/plane	1. Financial rewards as sole pharmacists in small towns 2. High income/Good salary/High earning potential 3. Financial aid from the state 4. Lower tuition fees at university for rural career 5. Tax concessions 6. Owning a pharmacy in rural area (as an Economic aspect) 7. Financial support/incentives for rural return of service/loan forgiveness programs 8. Funding incentive (e.g. scholarship, continuing professional development allowance) 9. Signing bonus, license fees paid, or similar benefits 10. Cost of living/affordability 11. Housing availability/affordability 12. Suitable accommodation 13. Higher level of debts/return-of-service commitments 14. Financial risk 15. Administrative expenses 16. Regulatory requirements 17. Better job security/ permanent fulltime employment 18. Resources in practice environment (good stock control, reduced access to services, logistical issues) 19. Rural scholarship 20. Availability of the costs of relocation 21. Cost of locums	1. Diverse work experience 2. Independency or autonomy of practice 3. Expanded scope of practice 4. Job/professional satisfaction 5. Access to continuing professional education/development 6. Rural career exposure/past employment 7. Internship in a rural area 8. Rural exposure in undergraduate pharmacy education 9. More than six years of practice experience 10. Greater job opportunities/career ladders 11. Professional isolation	1. Perception of being a good fit for rural practice 2. Satisfaction with professional aspect of working in a rural community 3. Confidence in providing healthcare services 4. Relationships/communication with co-workers or other health professionals 5. Working as part of multidisciplinary team/ getting along and working well together 6. Good work environment 7. Access to locum support 8. Supervision/preceptor/supervisor 9. Peer support 10. Good practice experience/ career opportunities 11. Good pace of work on the job 12. Ability to practice as desired 13. Good working hours 14. Availability of coverage and backup 15. Accessible to GP services/medical health care 16. Work/life balance (ability to take leave) 17. Availability of staff 18. Being employed as hospital pharmacist 19. Clinical and administrative workload	1. Serving health needs in the rural community/helping people 2. Feeling of being valued by the rural communities/sense of belonging 3. Sense of being appreciated by community/community recognition 4. Connection to community 5. Helping to develop rural areas 6. Starting a business ‘with friends’ 7. Communities are friendly and supportive of each other 8. Future of community looks very positive over next 5 years 9. Community attractiveness 10. Health care a major part of local economic development 11. Positive relationships/ communication with customers 12. Sense of loyalty to the pharmacy 13. Privacy/separation between personal and professional roles in small rural communities 14. Image/perception of rural health care 15. Differences in health issues compared to metropolitan areas

### Geographic and family-related factors

Geographic and family-related factors were identified in nine studies as the main contributors of recruitment and retention of rural pharmacist practice. Of these, the most common enablers were having a rural origin or currently living in rural areas, being married, having a spouse or partner, and with children or having a family [6, 7, 15, 32, 33, 36, 37]. This was closely followed by the rural lifestyle, quality of life, or life satisfaction associated with living in rural areas, or the family-friendly environment that a rural life offered [15, 31, 33, 37, 40].

Other less common factors positively associated with rural pharmacist workforce recruitment and retention were access to good quality schools or education system in rural areas [31], better access to recreational, physical and sporting opportunities [32], and size of the community not being too large and not too small [7, 40]. Additional, yet minor, enablers included the pharmacist being aged between 35 and 54 years [33], the perception of better opportunities for family members [33], a desire to return to their hometown [7], or other personal reasons, such as a desire to be independent from extended family or seeking adventure experiences [32].

Barriers identified from these studies included having less access to cultural and social activities, including facilities that enable such activities to occur [32, 36, 38], personal and social isolation, such as distance from or fewer relationships with friends, family and partners [15], and fear of unfamiliarity of rural and remote regions [15]. The study conducted in the Ukraine [38] showed that final year students who had a desire to work as rural pharmacists had higher expectation levels regarding the living conditions they would be working in than students who sought to work in urban areas. For example, the availability of public transport, public media, and internet access were key factors that these students consider when seeking to work in rural areas. In addition, these same students had higher levels of importance placed on the availability of public communication and cultural events within rural contexts than students who sought to work in urban areas.

### Economic and resources factors

Another main theme and group of factors associated with rural pharmacist workforce recruitment and retention were economics and resources, which were reported in 10 of the 13 studies identified. Financial rewards were considered positive factors, which comprised of a higher income or salary [7, 15, 32, 33, 36, 38, 39], and financial incentives and other benefits, including funding support from the government, specific remuneration packages and other contractual agreements [7, 15, 32, 37, 39, 40]. Other less common enablers included the waiving of loan debt (loan forgiveness) associated with completing undergraduate pharmacy programs [32, 34], low cost of living in rural areas [32, 37] and housing affordability or availability [37, 39].

Conversely, in a study from Australia [6], receiving a student rural scholarship was less likely to be associated with longer-term rural pharmacist practice, indicating that rural practice awareness may not always translate into rural recruitment or retention. In the study from the Ukraine [38], financial risk associated with rural pharmacy ownership acted as a disincentive for pharmacist students to enter rural areas, and despite attracting government financial aid, rural pharmacy ownership were associated with high levels of financial risk [38]. Final year pharmacy students intending to practice rurally upon graduation, had a higher level of expectation regarding being provided with financial support to relocate than students who sought to work in urban areas [38]. Thus, financial support may act as an incentive among those students who are seeking to work rurally. In addition, in the Australian study by Hays et al. [15], a lack of available resources, such as stock control, access to services, logistical delays, was identified as a disincentive to practice rurally amongst qualified pharmacists.

### Scope of practice or skills development factors

Scope of practice or skills development factors were reported to either encourage or discourage pharmacists to stay in rural practice settings. Three studies reported that having diverse work experience, such as being employed by different pharmacy providers and/or at various geographic places and across one’s career [36], expanded scope of practice (particularly in filling health services gaps in very remote areas without a full time doctor) [15] or having independence or autonomy of practice acted as an incentive for pharmacists to practice rurally [38]. Rural career exposure, including having placement and/or vocational assignment among pharmacy graduates [7], being trained or past employment in rural areas were positively associated rural pharmacist workforce recruitment and retention [6, 37, 40]. Other enablers were that those with between 6 and 24 years of practice experience were more likely to enter rural settings, and rural practice providing greater career opportunities [33].

Adequate access to continuing professional education or career development was also considered as an enabler, when available to pharmacists [6, 15, 32, 35, 37, 39]. The Ukraine study [38] highlighted that final year students desiring to work as rural pharmacists were more likely to expect greater availability of and accessibility to continuing education than other students, which if available, may further incentivise working in rural areas. In Australia, feelings of professional isolation were identified as another important barrier to rural pharmacist workforce recruitment and retention [15, 39].

### Practice environment factors

Many of the reviewed articles highlighted practice environment factors that had a propensity to influence rural pharmacist workforce recruitment and retention. Enablers varied, such as a positive work environment [39], the perception that rural practice was a good fit for the individual [35], or that the rural job provided a better pace of work than provided elsewhere [32]. Other enablers included increased confidence in providing healthcare services [35], having adequate locum support [33, 39], good relationships and communication with co-workers or other health professionals [31, 37], the ability to work as part of multidisciplinary team [15] and better job security in rural settings [15]. Final year students in the Ukraine, who had a desire to work as rural pharmacists, had lower expectations regarding the accessibility of medical health care than those students seeking to work in urban areas [38]. Thus, these findings suggest that students seeking to work as pharmacists in rural areas may be more tolerant of the limited accessibility of rural medical healthcare.

Although considered an enabler by some studies [33, 39], the most common barrier was limited access to locum pharmacists for support or to cover periods of leave [15, 36, 37, 39]. This was followed by staff shortages, such as a lack of technical, adjunct and retail support staff [15, 31], limited access to senior pharmacists to supervise junior pharmacists [39] and lack of peer support [36]. A shortage of primary medical services was found to be likely associated with pharmacist shortage in rural settings [35, 37]. Other study-specific factors that negatively impacted the retention of a rural pharmacist workforce were being employed as a hospital pharmacist, who were almost four times less likely to be working in a rural area [6], and dissatisfaction with current practice [39]. Commonly reported areas of dissatisfaction for hospital pharmacists included high clinical and administrative workloads, potential conflict in the workplace [39], or experiencing an unhealthy work-life balance given that pharmacists were unlikely to take leave when working in rural settings [39].

### Community and practice support factors

The final factor was the community and practice support factor, that captures the positive elements of the rural environments supporting rural pharmacist workforce recruitment and retention. These elements include contributing and serving the health needs in the community [7, 15, 36] and the fulfilment and enjoyment of helping rural people, communities, or helping to develop rural areas [38]. In addition, feeling a sense of belonging to a rural community [15, 33, 36, 39], building rapport with and having a good relationship with customers [15, 37, 39], and feeling valued or needed as an essential member of the rural community [7, 15, 36, 38] were all identified as exerting a positive influence upon rural pharmacist workforce recruitment and retention. Other enablers were community connections, either historical or familial [36], a friendly and supportive community [15, 31], and good relationships and collaboration between community health professionals or between health care providers and other sectors of community [31].

A barrier identified was the lack of privacy in small rural communities. Further, a negative image of rural health care (e.g., underestimation of the practice facilities, logistics pertaining to running a pharmacy, and that rural communities are somehow not as progressive as metropolitan areas) was also identified as a barrier to rural pharmacist practice by Harding et al. [37]. Conversely, some study participants [15] felt that the negative perceptions should be mitigated or at least the views concerning rural practice improved.

## Discussion

The purpose of this review was to systematically and comprehensively identify the factors associated with rural pharmacist workforce recruitment and retention. In addition, it was also to better understand these key factors in order to inform more effective interventional strategies that may mitigate against the pharmacist workforce shortfall. This review yielded 13 study reports, which highlighted the complexity of rural pharmacist workforce recruitment and retention and provided insights into five overarching themes. Geographic and family-related factors, economic and resource factors, scope of practice or skills development factors, practice environment factors, and community and practice support factors were identified. It is this range of heterogeneous factors that encompass the characteristics and parameters that impact on the rural pharmacist workforce recruitment and retention and are explored further here.

Although pharmacists play a unique and critical role in health care, particularly in rural and remote settings, it was noted that a number of commonalities exist with other health professions with regard to geographical factors associated with recruitment into rural and remote settings. Pharmacists have been shown to share similar needs and desires with medical, nursing, and other allied health professionals in terms of spousal employment, good quality schools for children, the size of the community, and access to recreational, physical, and cultural opportunities [3, 29, 41].

Another common factor is rural origin of the pharmacist, which is found to have a positive impact on rural practice decisions among pharmacists as well as general practitioners [42] and professionals across different healthcare fields [43, 44]. This may be due to familiarity with rural settings that foster an appreciation for the quality of life, facilitating social connection and integration into the community that a rural lifestyle can provide. Conversely, it is noteworthy to mention that, in one study, students receiving a rural scholarship, who were more likely to have come from a rural background, might not necessarily translate into motivation to remain rurally for pharmacist practice upon graduation [6]. Nevertheless, those health professionals from a rural background are more likely to decide to work and remain in rural practice for longer than their urban counterparts and this may be based more on individual choice rather than a scholarship [4, 44, 45].

Similarly, prior rural exposure was suggested to be the main factor influencing recruitment and retention among health practitioners [4, 44]. Increased rural experience and exposure through education and training (e.g., rural teaching and learning sites, rural clinical placements, or integration of rural content in curriculum) have been suggested as effective strategies in improving recruitment and retention of healthcare workforce [42, 44, 46]. This approach might also be effective for the pharmacist profession [2, 47]. The resultant familiarity with the rural setting might aid in meeting their needs, as identified in this review, especially regarding connection and integration with other healthcare professionals, and with the community at large.

Further, when examining other factors such as economic and resource factors, it is noted that again, common factors such as salary, cost of living, housing affordability, relocation support, and other financial benefits were shared considerations among all health professions, including dentists, nurses, and doctors [1, 3, 29, 44]. Of interest, the economic and resource factors exclusive to pharmacists when compared to dental, medicine and nursing professionals are concerned with limited practice resources and the financial risk of owning one’s own pharmacy. Also unique to rural pharmacies were government remuneration, benefits, and financial incentives [2, 48]. In a number of countries, governments recognise the additional financial burden of maintaining a pharmacy in rural areas and, in some jurisdictions, there are additional allowances to support pharmacist to open businesses or facilitate succession planning to ensure long term viability [48]. In addition, financial incentives, such as student loan repayment schemes, country-specific education loan forgiveness programs (e.g. United States), higher salaries, and financial remuneration, have also been recommended and implemented across countries and regions [44, 46]. However, such financial strategies might not always be effective as extrinsic motivators, such as income [49]. For example, while financial strategies may help initially to attract healthcare professionals to rural areas, including pharmacists, other strategies also need to address their intrinsic needs, such as personal and professional recognition, for more effective retention [49].

Pharmacists are health professionals with unique knowledge and skill sets, and this was evident when examining the scope of practice or skills development factors that are considered vital for considering rural practice. Despite their unique professional practice needs, a number of parallels were observed with the nursing and medical professions in terms of rural recruitment and retention. All groups shared a common focus on the need for and consideration of professional development and opportunities for career progression in rural practice contexts. In addition, aspects such as independence or autonomy within practice are considered important among all health professional groups along with the need for, or responsibility of, teaching or mentoring. Of interest, nurses shared a commonality with pharmacists regarding past employment in rural areas being positively associated with rural recruitment, however this finding was not observed among medical professionals [1, 3, 29].

Similarly, dental practitioners, who see increased clinical and administrative experience as having a positive influence [44], pharmacists tend to regard the expanded scope of practice or experience as a pull factor of rural practice. When pharmacists’ capacity is generally underutilised, it leads to possible frustration and dissatisfaction, the opportunities to extend their practice scope and experience in rural settings is arguably a motivator [50–52]. An ability to engage in various health services, especially through mutual support and to collaborate with GPs and other healthcare professionals, further contributes favourably toward their skill development as well as professional self-esteem [50–53]. However, expanding pharmacy practice in rural context faces several challenges at policy, rural health providers and personal levels, including government and funding support, practice environment, pharmacist capacity and community uptake [53]. These highlight the essential need for multifaceted strategies to overcoming rural pharmacist workforce challenge that will, in turn, lead to improved health outcomes for rural and remote communities.

Akin to scope of practice or skills development factors, practice environment factors were relatively unique to rural pharmacist workforce recruitment and retention. Although there remain some similarities with factors associated with nurse recruitment and retention in rural settings, such as having a positive work environment [3], many key elements were exclusive to the pharmacist workforce. These included the need for good inter- and intra-disciplinary relationships and communication as well as higher levels of job security that employment in rural settings offered [15, 31, 37].

Internships in rural areas were observed as positive, given they may be readily available, but supervision may be impacted by the limited financial incentive or capacity and professional desire to provide trainee supervision or peer support within rural settings [36, 39]. However, if an internship can be achieved, then rural retention is improved [6, 7]. It is noteworthy that, although job opportunities and security were evident in rural settings, a deficit remains in population of pharmacists that can or are willing to undertake rural employment. This challenge is further demonstrated through limited access to locum pharmacists to support current pharmacist who may desire or need personal or vacation leave and will be a consideration regarding the accepting of a rural pharmacist role or employment [15, 36, 37, 39].

Lastly, there were again similarities with other health professions in that the motivations for rural practice were for fulfillment and enjoyment of helping others, but also that sense of reciprocity that occurs between health professionals and community members [1, 3, 41]. In addition, the elements that impact pharmacist workforce recruitment and retention were shown to be linked to the sharing of community experience with other health professionals, an associated sense of belonging that comes from building rapport with customers and feeling valued as a member of the wider rural community [7, 15, 33, 36–39, 41]. Although similarities and positive shared experiences among the various health professions exist, pharmacists, much like their medical counterparts, also desire a level of anonymity within rural contexts [35, 36]. In addition, the profession remains challenged by a negative image of rural health care when considering a rural career, such as stereotypes of isolation, prohibitive access to metropolitan centres, and stigmatisation of place [37, 54], although some pharmacists felt that the views concerning rural places and the practices in which they occur have been improved [15].

Overall, factors influencing the rural pharmacist workforce recruitment and retention were found to be complex and multi-faceted, encompassing individual, professional, structural, social, and economic dimensions. A comprehensive and dynamic theoretical framework of these contributing factors is required to be developed if we are to meet the growing need of this essential link in the health professional chain. Such an endeavour must focus on the enablers and barriers of rural pharmacist workforce recruitment and retention, which are both modifiable and non-modifiable, while also recognising each factor’s interrelation and interaction. This approach would enable the design and implementation of effective interventions to inform future opportunities and research to improve recruitment and retention, service efficiency, and sustainability. In addition, to ensure well-tailored responses, special attention should be given to those push-pull factors that are considered important to pharmacists, which have far-reaching long-term effects on the rural pharmacy workforce.

## Limitations

For practical reasons, this review was restricted to peer-reviewed empirical evidence published in English; however, there is potential for citation bias due to the inclusion of hand searching reference lists to identify additional relevant studies. Secondly, rural pharmacist practice around the world is nuanced by a myriad of contextual factors - education, registration and licencing, government funding and subsidisation, the need or capacity to own a rural pharmacy, and geographic distribution of populations - that may make it difficult to make generalisations across the international population. Due to the heterogeneity across research articles in terms of study design, hypotheses, research questions, methodology, outcome measures, and findings, we were unable to perform meta-analysis and other sensitivity analyses to provide quantitative estimates. In addition, aside from the study within the Ukrainian context, all other studies represent middle to high-income countries, which may also limit the generalisability of the outcomes of this analysis of rural pharmacist workforce recruitment and retention factors to other settings.

## Conclusions

While there are commonalities between pharmacists and other healthcare professionals, our review highlights that rural practice motivators for pharmacists are linked to both personal and professional satisfaction. Specifically, their personal satisfaction is influenced by the extent to which the rural setting caters to their individual and familial needs, especially in terms of lifestyle, education, recreation, and community support. Their professional satisfaction is mostly associated with opportunities for continuing career development, enhanced practice scope and experiences, positive inter- and intra-disciplinary relationships, and satisfactory financial benefits. These factors should be taken into consideration when developing interventional strategies to resolve rural pharmacist workforce recruitment and retention shortfalls s. As such, a multi-component approach that considers and targets these factors would be highly recommended.

## Supplementary information

**Additional file 1.**

**Additional file 2.**

**Additional file 3.**

**Additional file 4.**

## Data Availability

Not applicable.
