# Correction to: Factors contributing to the recruitment and retention of rural pharmacist workforce: a systematic review

**DOI:** 10.1186/s12913-021-07175-9

**Published:** 2021-11-16

**Authors:** Daniel Terry, Hoang Phan, Blake Peck, Danny Hills, Mark Kirschbaum, Jaclyn Bishop, Kehinde Obamiro, Ha Hoang, Hoang Nguyen, Ed Baker, David Schmitz

**Affiliations:** 1 School of Health, Federation University Australia, Victoria, Australia; 2 Menzies Institute for Medical Research, University of Tasmania, Tasmania, Australia; 3 School of Social Sciences, University of Tasmania, Tasmania, Australia; 4 Pharmacy Board of Australia, Melbourne, Australia; 5 Western Alliance, Warrnambool, Victoria Australia; 6 Centre for Rural Health, University of Tasmania, Tasmania, Australia; 7 Wicking Dementia Research and Education Centre, University of Tasmania, Tasmania, Australia; 8 Center for Health Policy, Boise State University, Boise, ID USA; 9 Department of Family and Community Medicine, University of North Dakota, Grand Forks, USA

**Correction to: BMC Health Serv Res 21, 1052 (2021)**

**https://doi.org/10.1186/s12913-021-07072-1**

Following the publication of the original article [1], the authors’ affiliation should be corrected.

The authors’ affiliation has been updated in this correction article and the original article [1] has been corrected.
