# Supplementary material for: Factors contributing to the recruitment and retention of rural pharmacist workforce: a systematic review
Source: BMC Health Serv Res. 2021 Oct 5;21:1052. doi: 10.1186/s12913-021-07072-1 (PMC8493699; doi:10.1186/s12913-021-07072-1)
Supplement: Supplementary file 2 — Additional file 2. [file 12913_2021_7072_MOESM2_ESM.docx]

Additional File 2: *List of factors based on five major themes according barrier or enablers*

| **Enabler or barrier** | **Geographic (and family-related) factors** | **Economic/resources** | **Scope of Practice/Skills Development** | **Practice Environment** | **Community/practice support** |
| --- | --- | --- | --- | --- | --- |
| **Enablers** | - Having a family  - Good place to raise a family  - Family-friendly environment | - Financial rewards as sole pharmacists in small towns | - Diverse work experience | - Perception of being a good fit for rural practice  - Satisfaction with professional aspect of working in a rural community | - Serving health needs in the community  - Enjoys helping people  - Being able to assist in solving problems; being accessible  - Enjoyment of helping people  - Serving health needs in the community  - Serving health needs in the community |
|  | - Sociodemographic: being married, had children living at home, 35 and 54 years | - High income  - Good salary  - Good salary  - Good salary  - Good salary | - Independency or autonomy of practice | - Confidence in providing healthcare services | - Feeling of being valued by the rural communities  - Sense of belonging to the communities  - Feeling of being needed |
|  | - Lifestyle  - Rural lifestyle  - Satisfaction with personal aspect of living in the community | - Financial aid from the state  - Financial aid obligations/loan forgiveness | - Expanded scope of practice | - Positive relationships/ communication with co-workers or other health professionals  - Positive relationships/  communication with co-workers or other health professionals  - Friendly pharmacy staff/colleagues  - Interprofessional collaborative practice | - Community connections, either historical or family connection |
|  | - Lived in rural areas  - Rural origin  - Lived in rural areas/rural childhood  - Lived in rural areas  - Rural background | - Lower tuition  fees at university  - Tax concessions | - Job (professional) satisfaction  - Job (professional) satisfaction  - Job (professional) satisfaction | - Working as part of multidisciplinary team | - Helping to develop rural areas |
|  | - Desire to return to hometown | Owning a pharmacy  Owning a pharmacy in rural area | - Access to continuing professional education/development  - Access to continuing professional education/development | - Better job security/ permanent fulltime employment | - starting a business ‘with friends’ |
|  | - Currently living in or near the community  - Family in a rural area  - Spouse or partner with a rural background | - Financial support/incentives from the state government for students for return of service  - Financial incentives  - Financial incentives  - Funding sources and incentive programs e.g. scholarship, continuing professional development allowance | - Being trained in rural areas  - Past employment in a rural area  - Internship in a rural area  - Rural career exposure  - Placement/vocational assignment in rural areas | - Good work environment | - Community health professionals get along and work well together |
|  | - Desire of being independent from extended family or adventure  experiences | - Benefits: signing bonus, license fees paid, or similar benefits  - High earning potential | - 6-24 years of practice | - Access to locum support  - Access to locum support | - People in community are friendly and supportive of each other |
|  | - Proximity to family  - Owning a pharmacy in rural area | - Cost of living  - Cost of living/affordability | - Greater job opportunities | - Supportive preceptor/supervisor | - Health care providers and other sectors of community work well together |
|  | - good transport connections | - Housing availability/affordability  - Suitable accommodation |  | - Good practice experience/ career opportunities | - Future of community looks very positive over next 5 years |
|  | - Good public school system | - Higher level of debts/return-of-service commitments  - Higher levels of debts |  | - Good pace of work on the job | - Community attractiveness |
|  | - Better access to sports and physical activities  - Better recreational opportunities (/or cultural activities) |  |  | - Ability to practice as desired | - Health care a major part of local economic development |
|  | - Size of community (not too large)  - Size of community (not too small/too large) |  |  | - Better working hours | - Positive relationships/ communication with customers  - Positive relationships/ communication with customers  - Positive relationships/communication with customers |
|  | - Better opportunities for family members |  |  | - Better availability of coverage and backup | - Sense of belonging to community  - Sense of belonging to community  - Sense of belonging to community |
|  |  |  |  |  | - Sense of loyalty to your pharmacy |
|  |  |  |  |  | - Sense of being appreciated by community/community recognition  - Sense of being appreciated by community |
|  |  |  |  |  | - Community recognition  - Community recognition |
|  |  |  |  |  | - Multiculturalism: Many cultures existing in 1 community |
| **Barriers** | Owning a pharmacy in rural area | Owning a pharmacy  Owning a pharmacy in rural area | -Lack of professional development opportunities/career ladders  - Lack of professional development opportunities/career ladders  - Lack of professional development opportunities/career ladders | - Lack of peer support | - Lack of social and cultural facilities |
|  | - Fear of not having family accompany them | - Financial risk  - Financial risk | - Lack of face-to-face continuing professional education access | - Lack of locum support  - Limited availability of locums  - Limited access to locums  - Limited access to locums | - Lack of privacy: lack of separation between personal and professional roles in small rural communities  - Lack of privacy: lack of separation between personal and professional roles in small rural communities |
|  | - Lack of cultural activities | - High administrative expenses | - Placement/vocational assignment in rural areas | - Cost of locums | - Lack of personal contacts of the same age |
|  | - Metropolitan-based family and social ties | - Regulatory requirements | - Feeling of professional isolation  - Feeling of professional isolation | - Limited access to supervision | - People from outside might not, at first, recognise area’s positive qualities |
|  | - Personal & social isolation | - Large social obligation | -Rural (undergraduate) pharmacy education | - Shortage of GP services may link to pharmacist’s shortage  - Shortage of GP services | - Negative image of rural health care and/perceptions of rural health care |
|  | - Lack of relationships | - Challenge in resources in practice environment |  | - dedicating too much time to work | - Differences in health issues between metropolitan/regional and rural or remote regions |
|  | - Both parents born in Australia | - Received a rural scholarship |  | - Lack of staff  - Lack of staff | - Fear and unfamiliarity of rural and remote locations |
|  | - Less access to cultural and social activities |  |  | - Being employed as hospital pharmacist |  |
|  | - Spousal/partner satisfaction e.g. education, work, general |  |  | + high clinical and administrative workload |  |
|  |  |  |  | + Less of life balance (unable to take leave) |  |
|  |  |  |  | + Conflict in the workplace |  |
| **Other** | Students born in a rural area have:  - higher expectation of the accessibility of public media  - higher expectation of availability of public transport  Rural-intent vs urban-intent students have:  - higher expectation of the availability of public transport, public media and the internet  - higher expectation of the availability of public communication, cultural events | Rural-intent vs urban-intent students have:  - Higher expectation of the availability of the costs of relocation |  | Rural-intent vs urban-intent students have:  - higher expectation of the availability of continuing education  - lower expectation of accessible medical health care  - lower expectation of the availability of jobs | - Image of rural health care and /negative perceptions of rural health care need to be stopped |
