# Supplementary material for: Factors contributing to the recruitment and retention of rural pharmacist workforce: a systematic review
Source: BMC Health Serv Res. 2021 Oct 5;21:1052. doi: 10.1186/s12913-021-07072-1 (PMC8493699; doi:10.1186/s12913-021-07072-1)
Supplement: Supplementary file 3 — Additional file 3. [file 12913_2021_7072_MOESM3_ESM.docx]

| Additional File 3: *Checklist of factors based on five major themes* | | | | | | | |  |
| --- | --- | --- | --- | --- | --- | --- | --- | --- |
| **Study** | **Study design** | **Impact** | **Geographic (and family-related factors)** | **Economic/resources** | **Scope of Practice/Skills Development** | **Practice Environment** | **Community/practice support** | |
| Allan et al, 2007; Australia | *Qualitative* | Enabler | ✓ | ✓ | ✓ |  | ✓ | |
|  |  | Barrier |  |  | ✓ | ✓ | ✓ | |
| Allan et al, 2008; Australia | *Qualitative* | Enabler |  |  |  | ✓ |  | |
|  |  | Barrier |  |  |  | ✓ | ✓ | |
| Harding et al, 2006; Australia | *Qualitative* | Enabler | ✓ | ✓ | ✓ | ✓ | ✓ | |
|  |  | Barrier | ✓ |  | ✓ | ✓ | ✓ | |
| Hays et al, 2020; Australia | *Qualitative* | Enabler | ✓ | ✓ | ✓ | ✓ | ✓ | |
|  |  | Barrier | ✓ | ✓ | ✓ | ✓ | ✓ | |
|  |  | Other |  |  |  |  | ✓ | |
| Anzenberger, 2011; Ukraine | Mixed methods | Enabler | ✓ | ✓ | ✓ |  | ✓ | |
|  |  | Barrier | ✓ | ✓ |  | ✓ | ✓ | |
|  |  | Other | ✓ | ✓ |  | ✓ |  | |
| Smith, 2013; Australia | Mixed methods | Enabler |  | ✓ | ✓ | ✓ | ✓ | |
|  |  | Barrier |  |  | ✓ | ✓ |  | |
| Taylor et al, 2019; Australia | Mixed methods | Enabler | ✓ | ✓ | ✓ | ✓ |  | |
| Glasser, 2006; the USA | Quantitative | Enabler | ✓ |  |  | ✓ | ✓ | |
|  |  | Barrier |  |  |  | ✓ | ✓ | |
| Fleming and Spark, 2011; Australia | Quantitative | Enabler | ✓ |  | ✓ |  |  | |
|  |  | Barrier | ✓ | ✓ | ✓ | ✓ |  | |
| Pearson et al, 2010; Canada | Quantitative | Enabler | ✓ | ✓ | ✓ | ✓ |  | |
|  |  | Barrier | ✓ |  |  |  |  | |
| Woodend et al, 2004; Canada | Quantitative | Enabler | ✓ | ✓ | ✓ | ✓ | ✓ | |
| Ling et al, 2018; New Zealand | Quantitative | Enabler |  | ✓ |  |  |  | |
| Daniels et al, 2007; the USA | Quantitative | Enabler | ✓ | ✓ | ✓ |  | ✓ | |
