# Supplementary material for: Factors contributing to the recruitment and retention of rural pharmacist workforce: a systematic review
Source: BMC Health Serv Res. 2021 Oct 5;21:1052. doi: 10.1186/s12913-021-07072-1 (PMC8493699; doi:10.1186/s12913-021-07072-1)
Supplement: Supplementary file 4 — Additional file 4. [file 12913_2021_7072_MOESM4_ESM.docx]

Additional File 4: *List of factors based on five major themes according to author*

| **Study** | **Geographic (and family-related) factors** | **Economic/resources** | **Scope of Practice/Skills Development** | **Practice Environment** | **Community/practice support** |
| --- | --- | --- | --- | --- | --- |
| Allan et al, 2007; Australia | - Having a family | - Financial rewards as sole pharmacists in small towns | - Diverse work experience  - Lack of professional development opportunities/career ladders | - Lack of peer support  - Lack of locum support | - Serving health needs in the community  - Feeling of being valued by the rural communities; being able to assist in solving problems; being accessible  - Community connections, either historical or family connection  - Sense of belonging to the communities  - Lack of social and cultural facilities  - Lack of privacy: lack of separation between personal and professional roles in small rural communities |
|  |  |  |  |  |  |
| Allan et al, 2008; Australia |  |  |  | - Perception of being a good fit for rural practice  - Confidence in providing healthcare services  - Shortage of GP services may link to pharmacist’s shortage | - Lack of privacy: lack of separation between personal and professional roles in small rural communities |
| Anzenberger, 2011; Ukraine | Owning a pharmacy in rural area  - good transport connections  Owning a pharmacy in rural area  - Fear of not having family accompany them  - Lack of cultural activities  Students born in a rural area have:  - higher expectation of the accessibility of public media  - higher expectation of availability of public transport  Rural-intent vs urban-intent students have:  - higher expectation of the availability of public transport, public media and the internet  - higher expectation of the availability of public communication, cultural events | Owning a pharmacy  - High income  Owning a pharmacy in rural area  - Financial aid from the state  - Lower tuition  fees at university  - Tax concessions  Owning a pharmacy  - Financial risk  - High administrative expenses  - Regulatory requirements  - Large social obligation  Owning a pharmacy in rural area  - Financial risk  Rural-intent vs urban-intent students have:  - Higher expectation of the availability of the costs of relocation | Owning a pharmacy  - Independency or autonomy of practice | Owning a pharmacy  - dedicate too much time to work  Rural-intent vs urban-intent students have:  - higher expectation of the availability of continuing education  - lower expectation of accessible medical health care  - lower expectation of the availability of jobs | Owning a pharmacy:  - Enjoys helping people  - Feeling of being needed  Owning a pharmacy in rural area:  - Helping to develop rural areas  - Enjoyment of  helping people  - starting a business ‘with friends’  Owning a pharmacy in rural area:  - Lack of personal contacts of the same age |
|  |  |  |  |  |  |
|  |  |  |  |  |  |
| Glasser, 2006; the USA | - Good place to raise a family  - Good public school system |  |  | - Positive relationships/ communication with co-workers or other health professionals  - Lack of staff | - Community health professionals get along and work well together  - People in community are friendly and supportive of each other  - Health care providers and other sectors of community work well together  - Future of community looks very positive over next 5 years  - Community attractiveness  - Health care a major part of local economic development  - People from outside might not, at first, recognise area’s positive qualities |
|  |  |  |  |  |  |
| Harding et al, 2006 (Australia) | - Lived in rural areas  - Family-friendly environment  - Metropolitan-based family and social ties | - Financial support/incentives from the state government for students for return of service  - Cost of living  - Housing availability/affordability | - Job (professional) satisfaction  - Lack of face-to-face continuing professional education access  - Placement/vocational assignment in rural areas | - Positive relationships/  communication with co-workers or other health professionals  - Shortage of GP services  - Limited availability and cost of locums | - Positive relationships/ communication with customers  - Negative image of rural health care and/perceptions of rural health care |
|  |  |  |  |  |  |
| Hays et al, 2020 (Australia) | - Lifestyle  - Rural origin  - Personal & social isolation  - Lack of relationships | - Financial incentives  - Good salary  - Challenge in resources in practice environment | - Job (professional) satisfaction  - Expanded scope of practice  - Lack of professional development opportunities/career ladders  - Feeling of professional isolation | - Working as part of multidisciplinary team  - Better job security/ permanent fulltime employment  - Limited access to locums  - Lack of staff | - Serving health needs in the community  - Rural community  - Positive relationships/ communication with customers  - Sense of belonging to community  - Sense of loyalty to your pharmacy  - Sense of being appreciated by community/community recognition  - Differences in health issues between metropolitan/regional and rural or remote regions  - Fear and unfamiliarity of rural and remote locations  - Image of rural health care and /negative perceptions of rural health care need to be stopped |
|  |  |  |  |  |  |
|  |  |  |  |  |  |
| Fleming and Spark, 2011 (Australia) | - Lived in rural areas/rural childhood  - Family in a rural area  - Spouse or partner with a rural background  - Both parents born in Australia | - Received a rural scholarship | - Access to continuing professional education/development  - Being trained in rural areas  - Past employment in a rural area  - Internship in a rural area  - Rural (undergraduate) pharmacy education | - Being employed as hospital pharmacist |  |
|  |  |  |  |  |  |
| Smith, 2013 (Australia) |  | - Good salary  - Financial incentives  - Suitable accommodation | - Job (professional) satisfaction  - Feeling of professional isolation  - Lack of professional development opportunities/career ladders | - Good work environment  - Access to locum support  - Dissatisfaction with current practice e.g.  + high clinical and administrative workload  + Less of life balance (unable to take leave)  + Conflict in the workplace  - Limited access to locums  - Limited access to supervision | - Positive relationships/communication with customers  - Sense of belonging to community |
|  |  |  |  |  |  |
| Taylor et al, 2019 (Australia) | - Rural lifestyle | - Funding sources and incentive programs e.g. scholarship, continuing professional development allowance | - Rural career exposure | - Good practice experience/ career opportunities  - Friendly pharmacy staff/colleagues  - Interprofessional collaborative practice  - Supportive preceptor/supervisor |  |
| Pearson et al, 2010; Canada | - Lived in rural areas  - Currently living in or near the community  - Better access to sports and physical activities  - Size of community (not too large)  - Desire of being independent from extended family or adventure experiences  - Less access to cultural and social activities | - Cost of living/affordability  - Benefits: signing bonus, license fees paid, or similar benefits  - Good salary  - Higher level of debts/return-of-service commitments | - Access to continuing professional education/development | - Good pace of work on the job  - Ability to practice as desired |  |
|  |  |  |  |  |  |
| Woodend et al, 2004; Canada | - Sociodemographic: being married, had children living at home, 35 and 54 years;  - Better opportunities for family members  - Satisfaction with personal aspect of living in the community | - High earning potential | - 6-24 years of practice  - Greater job opportunities | - Satisfaction with professional aspect of working in a rural community  - Access to locum support  - Better working hours  - Better availability of coverage and backup | - Sense of belonging to community  - Sense of being appreciated by community/ Community recognition |
| Ling et al, 2018; New Zealand |  | - Higher levels of debts |  |  |  |
| Daniels et al, 2007; the USA | - Size of community (not too small/too large)  - Proximity to family  - Better recreational opportunities (/or cultural activities)  - Desire to return to hometown  - Rural background  - Spousal/partner satisfaction e.g. education, work, general | - Good salary  - Financial aid obligations/loan forgiveness | - Placement/vocational assignment in rural areas |  | - Serving health needs in the community  - Community recognition  - Multiculturalism: Many cultures existing in 1 community |
|  |  |  |  |  |  |
